# A Perspective on Electrical Stimulation and Sympathetic Regeneration in Peripheral Nerve Injuries

**DOI:** 10.1089/neur.2023.0133

**Published:** 2024-03-04

**Authors:** Tina Tian, Amy M. Moore, Paul A. Ghareeb, Nicholas M. Boulis, Patricia J. Ward

**Affiliations:** ^1^ Medical Scientist Training Program, Emory University, Atlanta, Georgia, USA.; ^2^ Neuroscience Graduate Program, Laney Graduate School, Emory University, Atlanta, Georgia, USA.; ^3^ Department of Cell Biology, School of Medicine, Emory University, Atlanta, Georgia, USA.; ^4^ Department of Plastic Surgery, The Ohio State University Wexner Medical Center, Columbus, Ohio, USA.; ^5^ Division of Plastic Surgery, Department of Surgery, Emory University, Atlanta, Georgia, USA.; ^6^ Department of Neurosurgery, Emory University, Atlanta, Georgia, USA.

**Keywords:** axonal injury, axonal regeneration, neuroexcitation, peripheral nerve injury, regeneration

## Abstract

Peripheral nerve injuries (PNIs) are common and devastating. The current standard of care relies on the slow and inefficient process of nerve regeneration after surgical intervention. Electrical stimulation (ES) has been shown to both experimentally and clinically result in improved regeneration and functional recovery after PNI for motor and sensory neurons; however, its effects on sympathetic regeneration have never been studied. Sympathetic neurons are responsible for a myriad of homeostatic processes that include, but are not limited to, blood pressure, immune response, sweating, and the structural integrity of the neuromuscular junction. Almost one quarter of the axons in the sciatic nerve are from sympathetic neurons, and their importance in bodily homeostasis and the pathogenesis of neuropathic pain should not be underestimated. Therefore, as ES continues to make its way into patient care, it is not only important to understand its impact on all neuron subtypes, but also to ensure that potential adverse effects are minimized. This piece gives an overview of the effects of ES in animals models and in humans while offering a perspective on the potential effects of ES on sympathetic axon regeneration.

## Introduction: Electrical Stimulation in Animal Models

Peripheral nerve injuries (PNIs) are common and have a yearly incidence of >500,000^1^; however, adjunctive treatment options improving the inefficient innate regenerative ability of peripheral nerves are limited.^2^ Currently, there are no U.S. Food and Drug Administration (FDA)-approved treatment options for improving peripheral nerve regeneration post-PNI, although activity-based therapies, such as electrical stimulation (ES), have received decades worth of pre-clinical research to support its potential as a complementary therapeutic option. The use of electrical stimulators is approved by the FDA; however, they are approved generally in the context of various pain states.^3–5^

The interest in ES as a potential method for reducing the functional decline exhibited in peripheral targets after PNI dates back as far as the early 1800s. For example, in 1841, denervated muscles of frogs that had experienced sciatic nerve injuries were found to contract with the application of ES and subsequently found to exhibit slower muscle atrophy.^6^ In 1945, ES was also found to slow the muscle atrophy of rat skeletal muscle after sciatic nerve injury.^7^

Since then, ES has been extensively studied in animal models with rodent models rising to the forefront. In rats, brief ES (1 h) at a 20-Hz frequency applied at the site of peripheral nerve repair improved axon regeneration across the coaptation site.^8–12^ This improved regeneration has also been observed with the use of nerve autografts, which entails two coaptation sites and larger nerve defects.^13^ ES has been found to enhance motor and sensory axon outgrowth into chronically denervated nerve stumps with improved distal target reinnervation in the context of delayed nerve repair in rats.^14–16^ Interestingly, enhancement was not observed when delayed nerve repair involved the use of a biodegradable conduit.^17^

In mice, ES has similarly been shown to enhance both motor and sensory regeneration after nerve repair.^18–21^ Additional sessions of ES performed for 3–4 weeks after the initial ES session have not been found to further enhance regeneration in both mice and rats.^13,18^ On the contrary, it is worthy to note that daily 1-h ES sessions for 1 week post-repair resulted in improved motor axon regeneration compared to a single application of ES at the time of nerve repair in rats,^22^ so the ideal paradigm appears to be unclear.

Conditioning ES is a protocol that involves stimulating a nerve prior to an injury.^23–29^ Conditioning ES has emerged as a potential method for improving outcomes in nerve transfer surgeries, which are relevant in cases such as brachial plexus injuries, proximal nerve injuries, spinal cord injury, and stroke. The idea of conditioning a nerve with ES stems from a conditioning lesion, which is a commonly used experimental technique that involves injuring the nerve with a “conditioning” lesion preceding a “test” lesion. Although still theoretical, a conditioning ES would allow for the donor nerve to be stimulated percutaneously, and non-injuriously, in the clinic at a specified time before the actual nerve transfer surgery to improve the efficiency and regenerative capacity of the donor nerve. Conditioning ES has displayed excellent results in the rat model, with motor and sensory recovery even surpassing those observed with perioperative ES^24,25^; however, more research is needed to solidify the feasibility and superiority of conditioning ES over perioperative ES given that this comparison has only been explored by a single lab group.

## Electrical Stimulation in Humans for Peripheral Nerve Injury

**FIG. 1.**
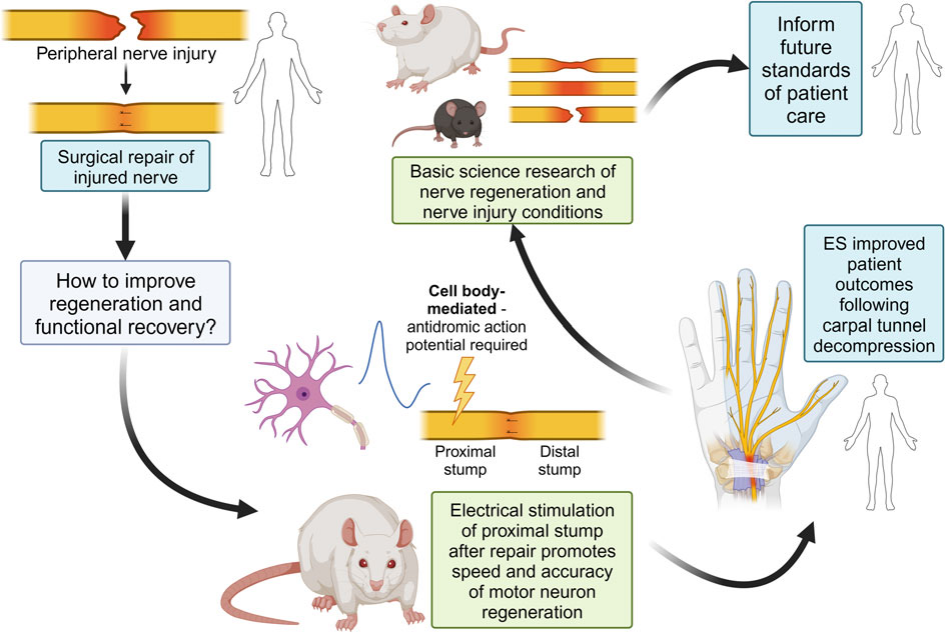
Cycle of how clinical scenarios inform basic science research, which then informs future standards of care for patients. The current standard of care of the treatment of peripheral nerve injuries relies on the slow and inefficient process of intrinsic axon regeneration with very few patients achieving full functional recovery. Therefore, in rats, 1 h of electrical stimulation (ES) of the proximal stump after surgical repair was found to be a viable method to promote motor axon regeneration into the distal stump.^8^ A clinical trial of ES in patients with severe carpal tunnel syndrome requiring surgical decompression resulted in improved functional recovery.^30^ On the basic science side, researchers continue to study different nerve injury scenarios and seek to understand and improve adjunctive therapeutic methods that will inform the future of patient care. Created with Biorender.com.

With the success of ES in accelerating axon regeneration and functional distal target reinnervation in animal models, several pilot clinical studies have been conducted in various patient populations (Fig. 1). Perioperative ES has shown promising results in clinical trials with studies showing improvement in both motor and sensory recovery. In one study, patients with severe carpal tunnel syndrome received 20 Hz of ES for 1 h with the stimulation intensity titrated to their maximal tolerance limit to the affected median nerve after carpal tunnel decompression surgery.^30^ This study found that ES resulted in more rapid improvement of motor and sensory function through nerve conduction studies as well as patient-reported outcome measures. This indicates that ES can be efficacious even after long-standing nerve injury present with severe carpal tunnel syndrome.

ES has also been studied as a potential adjunct treatment for facial nerve palsy, otherwise known as Bell's palsy.^31–36^ A randomized controlled study with patients diagnosed with mild-to-moderate Bell's palsy found that the addition of ES alongside prednisolone and/or acyclovir resulted in a significantly better recovery rate compared to those treated with prednisolone and/or acyclovir alone.^31^ Additionally, stimulation-induced blinking in patients with facial nerve palsy was found to reduce the negative ocular symptoms associated with facial nerve palsy in a preliminary study; however, the efficacy was likely limited to participants with less severe palsy.^33^ Three weeks of daily ES applied 4 weeks after facial nerve palsy onset was also found to improve functional facial movements and electrophysiological measures at the 3-month follow-up visit.^34^ On the other hand, the use of ES in the treatment of Bell's palsy in the acute phase (<30 days since onset) did not result in a statistically significant difference in terms of clinical effects.

Currently, the use of ES in humans is limited by the availability of FDA-approved intraoperative nerve stimulators. Clinical trials have often used external electrical stimulators such as the Grass SD9 and Echo Companion by Beac Biomedical Ltd, and some have used implantable stimulators such as the Itrel 7424 by Medtronic with varying stimulation parameters.^37–41^ With external stimulators, stimulation protocols proceeded for 1 h after the patient awakened from anesthesia, so the level of stimulation could be titrated to patient comfort.^30^ Implantable stimulators, however, have been developed for pain modulation. Over time, nerve stimulators have continued to improve in their ease of implantation in more hard-to-reach coaptation sites and their ability to minimize patient complications associated with an implanted stimulator.^42^

An absorbable hydrogel scaffold was recently developed and tested in rats.^42^ This hydrogel can also be formulated as a grounded gel, which can be used for percutaneous and open surgical approaches, with the formulation injected at the site of the injured nerve. This formulation stabilizes the electrodes, conforms to the surrounding anatomical features, and establishes a conductive path without electrode-nerve contact, making this method potentially attractive for the stimulation of nerves in anatomically challenging locations. The hydrogel led to improved motor and sensory recovery, increased muscle mass, and increased axonogenesis.

In light of these studies, the utility of ES to complement nerve repair in humans holds great potential for improving sensory and motor outcomes after PNI. However, very little is known about how ES affects the regeneration of sympathetic axons, which play major roles in thermoregulation, immune regulation, mitochondrial biogenesis in muscle, and more.^43–47^

## Anatomy of Peripheral Nerves

**FIG. 2.**
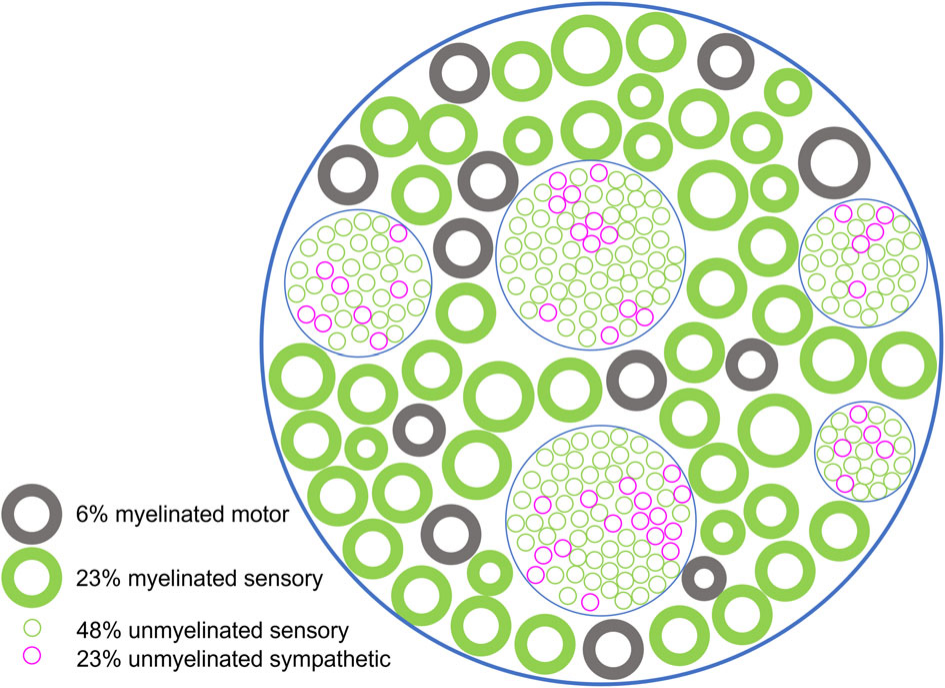
Anatomy schematic of the sciatic nerve. Cross-section diagram of the rat sciatic nerve. Unmyelinated sensory and post-ganglionic sympathetic neurons are clustered in Remak bundles. Figure adapted from Schmalbruch, 1986.^48^

When it comes to peripheral nerves, it is important to note that most of the axons are unmyelinated. Of the ∼27,000 axons in the rat sciatic nerve, only 29% are myelinated, consisting of 6% motor axons and 23% sensory axons.^48^ The rest of the nerve consists of 48% unmyelinated sensory axons and 23% unmyelinated sympathetic axons (Fig. 2).

Post-ganglionic parasympathetic axons, another important part of the autonomic nervous system that consists of unmyelinated axons, do not travel through the lumbar-level sciatic nerve.^49^ The remaining sections of this perspective piece will focus primarily on the unmyelinated sympathetic axons found nearly ubiquitously in peripheral nerves.

## Perspective on Electrical Stimulation for Sympathetic Axon Regeneration

Current research on the use of ES have been mostly focused on motor and sensory axon regeneration and their functional recovery. However, the paradigm that is widely used (20-Hz stimulation for 1 h at a stimulation intensity titrated to 1.5 × the intensity to elicit a muscle twitch in animal models and to patient comfort in humans) may not be at a high enough intensity to recruit sympathetic neurons into activity. Because of the small caliber and unmyelinated nature of sympathetic axons (in addition to other unmyelinated sensory axons), they will be recruited last into activity by ES. Larger caliber, myelinated axons are recruited first because of their lower input resistance attributed to having more parallel ion channels and nodes of Ranvier.^50^ Some experimental designs use stimulus intensities as high as 50 V to stimulate sympathetic neurons.^51,52^ In comparison, the stimulus intensity of ES in mouse models of PNI typically falls in the range of 3–4 V.^53^ Therefore, in order for the ES paradigm to recruit sympathetic neurons into activity, the stimulus intensity may not be in the realm of comfort for patients.

ES in itself is also non-specific in nature and can activate other cells around the axons that can influence axonal growth. Nerve growth factor (NGF), which binds to the tropomyosin receptor kinase A (TrkA) receptor, heavily influences sympathetic outgrowth, particularly sprouting. NGF is released by Schwann cells in response to ES, which may then increase sympathetic sprouting,^54^ a result that may hinder elongation and eventual functional reinnervation of distal targets. The presence of axonal sprouts, when coupled with elongation and the formation of new synapses, are critical for the reinnervation of distal targets after PNI; however, axonal sprouting alone without proper elongation may result in off-target effects and incomplete distal target reinnervation.

ES is thought to enhance axon regeneration through mechanisms that are also induced by a conditioning lesion.^55–57^ This conditioning lesion is believed to prime the neurons for regeneration; however, the regeneration of sympathetic axons in the sciatic nerve were shown to be inhibited by this technique.^57^ Thus, different axon types in the peripheral nerves may respond to activity-dependent interventions differently.

The inhibitory effect of the conditioning lesion on sympathetic outgrowth is inconclusive given that Navarro's group found that a conditioning lesion at the same site as a test lesion accelerated the growth of the sympathetic axons and shortened the time until most sweat glands became functionally reinnervated.^58^ Additionally, some studies have found that the superior cervical ganglia do respond positively to neuronal stimulation interventions such as conditioning lesions.^59^ Some of these incongruous findings may be attributable to the differing molecular profiles and heterogenous NGF requirements for target organ innervation of the sympathetic chain ganglia at different spinal levels.^60,61^ Therefore, further investigations are necessary to characterize how activity-based therapeutics such as ES affect different peripheral nerves.

Although several studies have investigated the impact of the conditioning lesion on sympathetic regeneration, the effects of ES on sympathetic regeneration and functional recovery over time after PNI are still unknown. Preliminary data suggest that ES at the very least is not improving the rate of sweat gland reinnervation and does not enhance sympathetic axon regeneration shortly after injury (Tian, unpublished). Additionally, it appears that activity-based therapies may also be detrimental to sympathetic regeneration overall, with specific stimulation of sympathetic neurons using bioluminescent optogenetics resulting in a decrease in the number of retrogradely-traced sympathetic neurons after sciatic nerve injury.^91^ Furthermore, conditioning ES significantly decreased the regeneration of sympathetic axons based on axon profile counts.^62–64^

The sympathetic nervous system innervates nearly every organ, including the skin and muscle, and is crucial for a vast array of normal bodily functions: from thermoregulation to blood pressure to immunoregulation to skin and muscle health.^43–47^ Loss of sympathetic innervation at the neuromuscular junction leads to synaptic instability, persistent muscle weakness despite maintenance of motoneuron innervation,^65^ and is a major driver of mitochondrial biogenesis in muscle.^66–68^ Thus, a need exists for treatments of PNI that promote the regeneration of all axons while avoiding potential adverse effects.

## Sympathetic Sprouting and Pain After Peripheral Nerve Injury

**FIG. 3.**
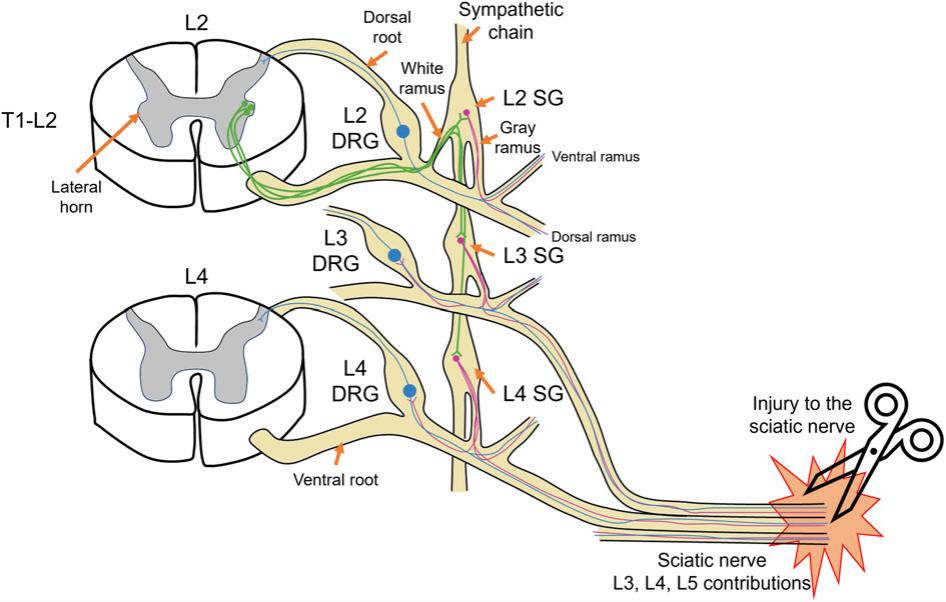
Post-ganglionic sympathetic neurons sprouting into the dorsal root ganglia (DRG) in response to peripheral nerve injury (PNI). The cell bodies of pre-ganglionic sympathetic neurons (green) are in the lateral horns of spinal cord levels T1–L2. Their projections travel along the ventral roots then through the white rami to innervate post-ganglionic sympathetic neurons (magenta) in the sympathetic chain. The pre-ganglionic neurons (green) can traverse several levels of the sympathetic chain before synapsing on post-ganglionic neurons. Most post-ganglionic axons exit the sympathetic ganglia (SG) through the gray rami and into the ventral and dorsal rami of the spinal nerves to innervate distal targets. Some will send projections to their adjacent DRG (sensory neurons in blue) to reach blood vessels and the surface of the DRG. These projections may sprout and contribute to neuropathic pain after PNI, such as that to the sciatic nerve, which in a mouse contains contributions mainly from the L3, L4, and L5 spinal nerve roots.^89,90^ For simplicity, only one neuron at each potential synapse is represented. Figure adapted from Xie and colleagues 2016.^73^

After PNI, patients may experience various degrees of neuropathic pain. This neuropathic pain is linked to sympathetic sprouting into the dorsal root ganglia (DRG) associated with the peripheral nerves that had been injured (Fig. 3).^69–73^ Sympathetic sprouting may include the formation of “baskets” around individual somas, particularly large-diameter neurons, and have also been observed in the DRGs of patients with neuropathic pain.^74,75^ In rats, a sympathectomy significantly reduces pain after spinal nerve ligation.^70^ Additionally, injury-induced spontaneous activity of primary sensory neurons at the level of the DRG have been implicated to play a critical role in the development of sympathetically maintained pain.^76^ It has been assumed that only DRG cells with a bursting discharge pattern or a discharge rate of >15 Hz may trigger sympathetic sprouting,^77^ with the large- and medium-sized DRG neurons most likely being the sites of sympathetic sprouting.^78–80^ Notably, the small unmyelinated DRG neurons typically do not exhibit these discharge patterns, more often exhibiting an irregular, low-frequency pattern instead.^80,81^

These findings in the pain literature may prove concerning for the use of ES, especially because evidence supports that early spontaneous activity is a trigger for persistent neuropathic pain after PNI,^76^ although this observation has not been systematically recorded in clinical populations. In many rodent models of PNI with ES, stimulation is applied at the time of injury, triggering a synchronized firing of mostly larger-caliber neurons. Synchronized cluster firing, defined as “more than three adjacently located DRG neurons that exhibit calcium transients within several continuous imaging frames,” is a characteristic found in DRG neurons that leads to spontaneous pain behavior.^82^ Cluster firing activity was found to be triggered by the sympathetic activity of fibers that had sprouted into DRGs after injury and is mediated by norepinephrine.

Further, ES has been shown to induce NGF release from Schwann cells.^54^ TrkA, which are high-affinity-NGF receptors, are found on nociceptors and sympathetic efferent fibers,^83^ and signaling through the NGF/TrkA pathway has been a target for treatment of pain.^84^ Transdermal ES after NGF administration to the hindlimbs of pigs resulted in a hyperalgesia, which was increased with higher-frequency stimulation (≥20 Hz) and was peaked at 3 weeks post-NGF administration.^85^ Pre-clinical evaluation of NGF and TrkA antagonism consistently prevented hyperalgesia and allodynia,^86,87^ but anti-NGF monoclonal antibodies in humans have shown mixed efficacy and lack long-term follow-up studies.^88^

## Conclusion

Despite decades of research in the viability of ES, the effects of this clinically relevant adjunctive treatment option on sympathetic regeneration remains unknown. Sympathetic axons comprise of nearly one fourth of axons in the sciatic nerve and account for many major bodily functions. Further, with the high likelihood that the current clinical paradigm is unable to recruit the small-diameter sympathetic fibers into activity, the effects of ES may be more dependent on factors released by surrounding cells. Therefore, optimization of the stimulation paradigm needs to be explored alongside the effects of activity-dependent therapeutics on the regeneration of different neuron types. And, finally, with the relationship between neuropathic pain and sympathetic sprouting, which also appears to be activity-dependent and related to the injury-induced spontaneous activity of larger cells, the potential adverse effects of ES need to be further examined. With this intervention gaining more traction in the clinical realm, it becomes imperative that the effects of ES on peripheral nerve regeneration must be studied in sympathetic neurons in peripheral nerves.

## Abbreviations Used

DRG: dorsal root ganglia
ES: electrical stimulation
FDA: U.S. Food and Drug Administration
NGF: nerve growth factor
PNI: peripheral nerve injury
TrkA: tropomyosin receptor kinase A

## References

[1] Taylor CA, Braza D, Rice JB, et al. The incidence of peripheral nerve injury in extremity trauma. Am J Phys Med Rehabil 2008;87(5):381–385; doi: 10.1097/PHM.0b013e31815e6370 18334923

[2] Scholz T, Krichevsky A, Sumarto A, et al. Peripheral nerve injuries: an international survey of current treatments and future perspectives. J Reconstr Microsurg 2009;25(06):339–344; doi: 10.1055/s-0029-1215529 19301234

[3] Anthony CL, Tora MS, Bentley JN, et al. Dorsal root ganglion stimulation for thoracic neuralgia: a report of six cases. Cureus 2019;11(5):e4615; doi: 10.7759/cureus.4615 31312542 PMC6615574

[4] Texakalidis P, Tora MS, Boulis NM. Neurosurgeons' armamentarium for the management of refractory postherpetic neuralgia: a systematic literature review. Stereotact Funct Neurosurg 2019;97(1):55–65; doi: 10.1159/000499476 30995653

[5] Keifer OP Jr, Diaz A, Campbell M, et al. Occipital nerve stimulation for the treatment of refractory occipital neuralgia: a case series. World Neurosurg 2017;105:599–604; doi: 10.1016/j.wneu.2017.06.064 28634063

[6] Reid J. On the relation between muscular contractility and the nervous system. Lond Edin Mon J Med Sci 1841;1:310.

[7] Hines HM, Melville E, Wehrmacher WH. The effect of electrical stimulation on neuromuscular regeneration. Am J Physiol 1945;144(2):278–283; doi: 10.1152/ajplegacy.1945.144.2.278

[8] Al-Majed AA, Neumann CM, Brushart TM, et al. Brief electrical stimulation promotes the speed and accuracy of motor axonal regeneration. J Neurosci 2000;20(7):2602–2608; doi: 10.1523/JNEUROSCI.20-07-02602.2000 10729340 PMC6772244

[9] Brushart TM, Hoffman PN, Royall RM, et al. Electrical stimulation promotes motoneuron regeneration without increasing its speed or conditioning the neuron. J Neurosci 2002;22(15):6631–6638; doi: 10.1523/JNEUROSCI.22-15-06631.2002 12151542 PMC6758126

[10] Brushart TM, Jari R, Verge V, et al. Electrical stimulation restores the specificity of sensory axon regeneration. Exp Neurol 2005;194(1):221–229; doi: 10.1016/j.expneurol.2005.02.007 15899259

[11] Keane GC, Pan D, Roh J, et al. The effects of intraoperative electrical stimulation on regeneration and recovery after nerve isograft repair in a rat model. Hand (N Y) 2022;17(3):540–548; doi: 10.1177/1558944720939200 32666827 PMC9112755

[12] Roh J, Schellhardt L, Keane GC, et al. Short-duration, pulsatile, electrical stimulation therapy accelerates axon regeneration and recovery following tibial nerve injury and repair in rats. Plastic Reconstr Surg 2022;149(4):681e–690e; doi: 10.1097/PRS.0000000000008924 PMC8969122 35139047

[13] Zuo KJ, Shafa G, Antonyshyn K, et al. A single session of brief electrical stimulation enhances axon regeneration through nerve autografts. Exp Neurol 2020;323:113074; doi: 10.1016/j.expneurol.2019.113074 31655047

[14] Huang J, Zhang Y, Lu L, et al. Electrical stimulation accelerates nerve regeneration and functional recovery in delayed peripheral nerve injury in rats. Eur J Neurosci 2013;38(12):3691–3701; doi: 10.1016/j.expneurol.2019.113074 24118464

[15] Xu C, Kou Y, Zhang P, et al. Electrical stimulation promotes regeneration of defective peripheral nerves after delayed repair intervals lasting under one month. PLoS One 2014;9(9):e105045; doi: 10.1371/journal.pone.0105045 25181499 PMC4152131

[16] Elzinga K, Tyreman N, Ladak A, et al. Brief electrical stimulation improves nerve regeneration after delayed repair in Sprague Dawley rats. Exp Neurol 2015;269:142–153; doi: 10.1016/j.expneurol.2015.03.022 25842267

[17] Han N, Xu CG, Wang TB, et al. Electrical stimulation does not enhance nerve regeneration if delayed after sciatic nerve injury: the role of fibrosis. Neural Regen Res 2015;10(1):90–94; doi: 10.4103/1673-5374.150714 25788926 PMC4357124

[18] Park S, Liu CY, Ward PJ, et al. Effects of repeated 20-Hz electrical stimulation on functional recovery following peripheral nerve injury. Neurorehabil Neural Repair 2019;33(9):775–784; doi: 10.1177/1545968319862563 31328654 PMC6693960

[19] English AW, Schwartz G, Meador W, et al. Electrical stimulation promotes peripheral axon regeneration by enhanced neuronal neurotrophin signaling. Dev Neurobiol 2007;67(2):158–172; doi: 10.1002/dneu.20339 17443780 PMC4730384

[20] Halevi AE, Schellhardt L, Snyder-Warwick A, et al. Understand the neuro-enhancing effects of electrical stimulation in a mouse model. J Am Coll Surg 2019;229(4):S232; 10.1016/j.jamcollsurg.2019.08.509

[21] Wariyar SS, Brown AD, Tian T, et al. Angiogenesis is critical for the exercise-mediated enhancement of axon regeneration following peripheral nerve injury. Exp Neurol 2022;353:114029; doi: 10.1016/j.expneurol.2022.114029 35259353 PMC12360107

[22] Koo J, MacEwan MR, Kang SK, et al. Wireless bioresorbable electronic system enables sustained nonpharmacological neuroregenerative therapy. Nat Med 2018;24(12):1830–1836; doi: 10.1038/s41591-018-0196-2 30297910

[23] Senger JB, Rabey KN, Morhart MJ, et al. Conditioning electrical stimulation accelerates regeneration in nerve transfers. Ann Neurol 2020;88(2):363–374; doi: 10.1002/ana.25796 32447758

[24] Senger JB, Chan AW, Chan KM, et al. Conditioning electrical stimulation is superior to postoperative electrical stimulation in enhanced regeneration and functional recovery following nerve graft repair. Neurorehabil Neural Repair 2020;34(4):299–308; doi: 10.1177/1545968320905801 32089098

[25] Senger JB, Chan KM, Webber CA. Conditioning electrical stimulation is superior to postoperative electrical stimulation, resulting in enhanced nerve regeneration and functional recovery. Exp Neurol 2020;325:113147; doi: 10.1016/j.expneurol.2019.113147 31837321

[26] Senger JL, Chan KM, Macandili H, et al. Conditioning electrical stimulation promotes functional nerve regeneration. Exp Neurol 2019;315:60–71; doi: 10.1016/j.expneurol.2019.02.001 30731076

[27] Senger JLB, Verge V, Macandili H, et al. Electrical stimulation as a conditioning strategy for promoting and accelerating peripheral nerve regeneration. Exp Neurol 2018;302:75–84; doi: 10.1016/j.expneurol.2017.12.013 29291403

[28] Senger JB, Verge VM, Chan KM, Webber CA. The nerve conditioning lesion: a strategy to enhance nerve regeneration. Ann Neurol 2018;83(4):691–702; doi: 10.1002/ana.25209 29537631

[29] Webber CA, Senger JL, Acton L, et al. Unlike A Conditioning Crush Lesion, Conditioning Electrical Stimulation Promotes Functional Sensory And Motor Nerve Regeneration In A Non-inflammatory Manner. Plast Reconstr Surg Glob Open 2020;8(4 Suppl):76; doi: 10.1097/01.GOX.0000667516.98514.94

[30] Gordon T, Amirjani N, Edwards DC, et al. Brief post-surgical electrical stimulation accelerates axon regeneration and muscle reinnervation without affecting the functional measures in carpal tunnel syndrome patients. Exp Neurol 2010;223(1):192–202; doi: 10.1016/j.expneurol.2009.09.020 19800329

[31] Kim J, Choi JY. The effect of subthreshold continuous electrical stimulation on the facial function of patients with Bell's palsy. Acta Otolaryngol 2016;136(1):100–105; doi: 10.3109/00016489.2015.1083121 26399994

[32] Yoo MC, Kim JH, Kim YJ, et al. Effects of electrical stimulation on facial paralysis recovery after facial nerve injury: a review on preclinical and clinical studies. J Clin Med 2023;12(12):4133; doi: 10.3390/jcm12124133 37373826 PMC10299525

[33] Mäkelä E, Venesvirta H, Ilves M, et al. Electrically induced blink for the prevention of ocular symptoms and blurred vision in patients with acute facial nerve palsy. Ear Nose Throat J 2021; doi: 10.1177/01455613211048576 34714168

[34] Tuncay F, Borman P, Taser B, et al. Role of electrical stimulation added to conventional therapy in patients with idiopathic facial (Bell) palsy. Am J Phys Med Rehabil 2015;94(3):222–228; doi: 10.1097/PHM.0000000000000171 25171666

[35] Alakram P, Puckree T. Effects of electrical stimulation on house-brackmann scores in early bells palsy. Physiother Theory Pract 2010;26(3):160–166; doi: 10.3109/09593980902886339 20331372

[36] Borschel G. Electrical Stimulation to Improve Recovery After Peripheral Nerve Injury. ClinicalTrials.gov; 2023. Available from: https://clinicaltrials.gov/study/NCT03996525 [Last accessed: October 4, 2023].

[37] Williams HB. A clinical pilot study to assess functional return following continuous muscle stimulation after nerve injury and repair in the upper extremity using a completely implantable electrical system. Microsurgery 1996;17(11):597–605; doi: 10.1002/(SICI)1098-2752(1996)17:11<597::AID-MICR6>3.0.CO;2-M 9514518

[38] Piccinini G, Cuccagna C, Caliandro P, et al. Efficacy of electrical stimulation of denervated muscle: a multicenter, double-blind, randomized clinical trial. Muscle Nerve 2020;61(6):773–778; doi: 10.1002/mus.26880 32249950

[39] Wong JN, Olson JL, Morhart MJ, et al. Electrical stimulation enhances sensory recovery: a randomized controlled trial. Ann Neurol 2015;77(6):996–1006; doi: 10.1002/ana.24397 25727139

[40] Power HA, Morhart MJ, Olson JL, et al. Postsurgical electrical stimulation enhances recovery following surgery for severe cubital tunnel syndrome: a double-blind randomized controlled trial. Neurosurgery 2020;86(6):769–777; doi: 10.1093/neuros/nyz322 31432080

[41] ElAbd R, Alabdulkarim A, AlSabah S, et al. Role of electrical stimulation in peripheral nerve regeneration: a systematic review. Plast Reconstr Surg Glob Open 2022;10(3):e4115; doi: 10.1097/GOX.0000000000004115 35317464 PMC8932473

[42] Srinivasan S, Gfrerer L, Karandikar P, et al. Absorbable Conductive Electrotherapeutic Scaffolds (ACES) for Enhanced Peripheral Nerve Regeneration and Stimulation. bioRxiv 2022; doi: 10.1101/2022.07.30.500547 37339635

[43] Gagnon D, Crandall CG. Sweating as a heat loss thermoeffector. Handb Clin Neurol 2018;156:211–232; doi: 10.1016/B978-0-444-63912-7.00013-8 30454591

[44] Grassi G. Role of the sympathetic nervous system in human hypertension. J Hypertens 1998;16(12 Pt 2):1979–1987; doi: 10.1097/00004872-199816121-00019 9886886

[45] Dibona GF. Sympathetic nervous system and the kidney in hypertension. Curr Opin Nephrol Hypertens 2002;11(2):197–200; doi: 10.1097/00041552-200203000-00011 11856913

[46] Elenkov IJ, Wilder RL, Chrousos GP, et al. The sympathetic nerve—an integrative interface between two supersystems: the brain and the immune system. Pharmacol Rev 2000;52(4):595–638. 11121511

[47] Besedovsky HO, Del Rey A, Sorkin E, et al. Immunoregulation mediated by the sympathetic nervous system. Cell Immunol 1979;48(2):346–355; doi: 10.1016/0008-8749(79)90129-1 389444

[48] Schmalbruch H. Fiber composition of the rat sciatic nerve. Anat Rec 1986;215(1):71–81; doi: 10.1002/ar.1092150111 3706794

[49] Crone C, Krarup C. Neurophysiological approach to disorders of peripheral nerve. Handb Clin Neurol 2013;115:81–114; doi: 10.1016/B978-0-444-52902-2.00006-0 23931776

[50] Llewellyn ME, Thompson KR, Deisseroth K, et al. Orderly recruitment of motor units under optical control in vivo. Nat Med 2010;16(10):1161–1165; doi: 10.1038/nm.2228 20871612 PMC5839640

[51] De Jonge A, Timmermans P, Van Zwieten P. Participation of cardiac presynaptic α 1-adrenoceptors in the bradycardiac effects of clonidine and analogues. Naunyn Schmiedebergs Arch Pharmacol 1981;317(1):8–12; doi: 10.1007/BF00506249 6116198

[52] Boerman E, Jenkins S, Ramos K, et al. Perivascular adipose and perivascular nerve dysfunction in inflammatory bowel disease is reversed after macrophage depletion. Physiology 2023;38(S1):5725808; doi: 10.1152/physiol.2023.38.S1.5725808

[53] Geremia NM, Gordon T, Brushart TM, et al. Electrical stimulation promotes sensory neuron regeneration and growth-associated gene expression. Exp Neurol 2007;205(2):347–359; doi: 10.1016/j.expneurol.2007.01.040 17428474

[54] Huang J, Ye Z, Hu X, et al. Electrical stimulation induces calcium-dependent release of NGF from cultured Schwann cells. Glia 2010;58(5):622–631; doi: 10.1002/glia.20951 19998481

[55] McQuarrie IG, Grafstein B, Gershon MD. Axonal regeneration in the rat sciatic nerve: effect of a conditioning lesion and of dbcAMP. Brain Res 1977;132(3):443–453; doi: 10.1016/0006-8993(77)90193-7 199316

[56] McQuarrie IG, Grafstein B. Effect of a conditioning lesion on optic nerve regeneration in goldfish. Brain Res 1981;216(2):253–264; doi: 10.1016/0006-8993(81)90128-1 7248777

[57] McQuarrie IG, Grafstein B, Dreyfus CF, et al. Regeneration of adrenergic axons in rat sciatic nerve: effect of a conditioning lesion. Brain Res 1978;141(1):21–34; doi: 10.1016/0006-8993(78)90614-5 624075

[58] Navarro X, Kennedy WR. The effect of a conditioning lesion on sudomotor axon regeneration. Brain Res 1990;509(2):232–236; doi: 10.1016/0006-8993(90)90547-o 2322820

[59] Shoemaker S, Sachs HH, Vaccariello S, et al. A conditioning lesion enhances sympathetic neurite outgrowth. Exp Neurol 2005;194(2):432–443; doi: 10.1016/j.expneurol.2005.02.023 16022869

[60] Furlan A, La Manno G, Lübke M, et al. Visceral motor neuron diversity delineates a cellular basis for nipple-and pilo-erection muscle control. Nat Neurosci 2016;19(10):1331–1340; doi: 10.1038/nn.4376 27571008

[61] Glebova NO, Ginty DD. Heterogeneous requirement of NGF for sympathetic target innervation in vivo. J Neurosci 2004;24(3):743–751; doi: 10.1523/JNEUROSCI.4523-03.2004 14736860 PMC6729267

[62] Tian T, Ward J. Neuronal activity-based therapies do not enhance sympathetic axonal regeneration. Mary Ann Liebert, Inc; New Rochelle, NY: 2022.

[63] Tian T, Harris A, SiMa HM, et al, Sympathetic axon regeneration is not enhanced by adjunctive stimulation therapies. MD/PhD Program, Emory University School of Medicine. Available from: https://ps-rc.org/meeting/program/2023/assets/EP18.pdf [Last accessed: February 18, 2024].

[64] Tian T, Harris A, Owyoung J, et al. Conditioning electrical stimulation fails to enhance sympathetic axon regeneration. bioRxiv 2023; doi: 10.1101/2023.02.03.527071 40317121

[65] Khan MM, Lustrino D, Silveira WA, et al. Sympathetic innervation controls homeostasis of neuromuscular junctions in health and disease. Proc Natl Acad Sci U S A 2016;113(3):746–750; doi: 10.1073/pnas.1524272113 26733679 PMC4725522

[66] Straka T, Vita V, Prokshi K, et al. Postnatal development and distribution of sympathetic innervation in mouse skeletal muscle. Int J Mol Sci 2018;19(7):1935; doi: 10.3390/ijms19071935 29966393 PMC6073285

[67] Geng T, Li P, Okutsu M, et al. PGC-1α plays a functional role in exercise-induced mitochondrial biogenesis and angiogenesis but not fiber-type transformation in mouse skeletal muscle. Am J Physiol Cell Physiol 2010;298(3):C572–C579; doi: 10.1152/ajpcell.00481.2009 20032509 PMC3353735

[68] Lin J, Handschin C, Spiegelman BM. Metabolic control through the PGC-1 family of transcription coactivators. Cell Metab 2005;1(6):361–370; doi: 10.1016/j.cmet.2005.05.004 16054085

[69] Ramer MS, French GD, Bisby MA. Wallerian degeneration is required for both neuropathic pain and sympathetic sprouting into the DRG. Pain 1997;72(1–2):71–78; doi: 10.1016/s0304-3959(97)00019-5 9272789

[70] Chung K, Lee BH, Yoon YW, et al. Sympathetic sprouting in the dorsal root ganglia of the injured peripheral nerve in a rat neuropathic pain model. J Comp Neurol 1996;376(2):241–252; 8951640 10.1002/(SICI)1096-9861(19961209)376:2<241::AID-CNE6>3.0.CO;2-3

[71] Lee BH, Yoon YW, Chung K, et al. Comparison of sympathetic sprouting in sensory ganglia in three animal models of neuropathic pain. Exp Brain Res 1998;120:432–438; doi: 10.1007/s002210050416 9655228

[72] McLachlan EM, Jänig W, Devor M, et al. Peripheral nerve injury triggers noradrenergic sprouting within dorsal root ganglia. Nature 1993;363(6429):543–546; doi: 10.1038/363543a0 8505981

[73] Xie W, Chen S, Strong JA, et al. Localized sympathectomy reduces mechanical hypersensitivity by restoring normal immune homeostasis in rat models of inflammatory pain. J Neurosci 2016;36(33):8712–8725; doi: 10.1523/JNEUROSCI.4118-15.2016 27535916 PMC4987440

[74] Ramer MS, Thompson SW, McMahon SB. Causes and consequences of sympathetic basket formation in dorsal root ganglia. Pain 1999;82(Suppl 6):S111–S120; doi: 10.1016/S0304-3959(99)00144-X 10491979

[75] Shinder V, Govrin-Lippmann R, Cohen S, et al. Structural basis of sympathetic-sensory coupling in rat and human dorsal root ganglia following peripheral nerve injury. J Neurocytol 1999;28:743–761; doi: 10.1023/a:1007090105840 10859576

[76] Zhang J-M, Strong JA. Recent evidence for activity-dependent initiation of sympathetic sprouting and neuropathic pain. Sheng Li Xue Bao 2008;60(5):617–627. 18958370

[77] Burchiel KJ. Effects of electrical and mechanical stimulation on two foci of spontaneous activity which develop in primary afferent neurons after peripheral axotomy. Pain 1984;18(3):249–265; doi: 10.1016/0304-3959(84)90820-0 6728494

[78] Wall P, Devor M. Sensory afferent impulses originate from dorsal root ganglia as well as from the periphery in normal and nerve injured rats. Pain 1983;17(4):321–339; doi: 10.1016/0304-3959(83)90164-1 6664680

[79] Burchiel KJ. Spontaneous impulse generation in normal and denervated dorsal root ganglia: sensitivity to alpha-adrenergic stimulation and hypoxia. Exp Neurol 1984;85(2):257–272; doi: 10.1016/0014-4886(84)90139-0 6745375

[80] Devor M, Janig W, Michaelis M. Modulation of activity in dorsal root ganglion neurons by sympathetic activation in nerve-injured rats. J Neurophysiol 1994;71(1):38–47; doi: 10.1152/jn.1994.71.1.38 8158237

[81] Xie Y, Zhang J, Petersen M, et al. Functional changes in dorsal root ganglion cells after chronic nerve constriction in the rat. J Neurophysiol 1995;73(5):1811–1820; doi: 10.1152/jn.1995.73.5.1811 7623082

[82] Zheng Q, Xie W, Lückemeyer DD, et al. Synchronized cluster firing, a distinct form of sensory neuron activation, drives spontaneous pain. Neuron 2022;110(2):209–220.e6; doi: 10.1016/j.neuron.2021.10.019 34752775 PMC8776619

[83] Vega J, Vazquez E, Naves F, et al. Immunohistochemical localization of the high-affinity NGF receptor (gp 140-trkA) in the adult human dorsal root and sympathetic ganglia and in the nerves and sensory corpuscles supplying digital skin. Anat Rec 1994;240(4):579–588; doi: 10.1002/ar.1092400415 7879909

[84] Hirose M, Kuroda Y, Murata E. NGF/TrkA signaling as a therapeutic target for pain. Pain Practice 2016;16(2):175–182; doi: 10.1111/papr.12342 26452158

[85] Obreja O, Kluschina O, Mayer A, et al. NGF enhances electrically induced pain, but not axon reflex sweating. Pain 2011;152(8):1856–1863; doi: 10.1016/j.pain.2011.04.002 21546161

[86] Jimenez-Andrade JM, Martin CD, Koewler NJ, et al. Nerve growth factor sequestering therapy attenuates non-malignant skeletal pain following fracture. Pain 2007;133(1–3):183–196; doi: 10.1016/j.pain.2007.06.016 17693023

[87] Guerios SD, Wang ZY, Boldon K, et al. Blockade of NGF and trk receptors inhibits increased peripheral mechanical sensitivity accompanying cystitis in rats. Am J Physiol Regul Integr Comp Physiol 2008;295(1):R111–R122; doi: 10.1152/ajpregu.00728.2007 18448607 PMC2494812

[88] Chang DS, Hsu E, Hottinger DG, et al. Anti-nerve growth factor in pain management: current evidence. J Pain Res 2016;9:373–383; doi: 10.2147/JPR.S89061 27354823 PMC4908933

[89] Laedermann CJ, Pertin M, Suter MR, et al. Voltage-gated sodium channel expression in mouse DRG after SNI leads to re-evaluation of projections of injured fibers. Mol Pain 2014;10:19; doi: 10.1186/1744-8069-10-19 24618114 PMC4007621

[90] Rigaud M, Gemes G, Barabas ME, et al. Species and strain differences in rodent sciatic nerve anatomy: implications for studies of neuropathic pain. Pain 2008;136(1–2):188–201; doi: 10.1016/j.pain.2008.01.016 18316160 PMC2700063

[91] Tian T, Ward PJ. Neuronal activity-based therapies do not enhance sympathetic axonal regeneration. The 39th Annual Symposium of the National Neurotrauma Society, including the AANS/CNS Joint Section on Neurotrauma and Critical Care. J Neurotrauma 2022;39(11–12):A-1-A-128; doi: 10.1089/neu.2022.29126.abstracts

